# Supplementary figures and images for: Large-scale mouse mutagenesis identifies novel genes affecting vertebral anatomy
Source: Mamm Genome. 2026 Feb 5;37(1):34. doi: 10.1007/s00335-025-10189-x (PMC12876460; doi:10.1007/s00335-025-10189-x)

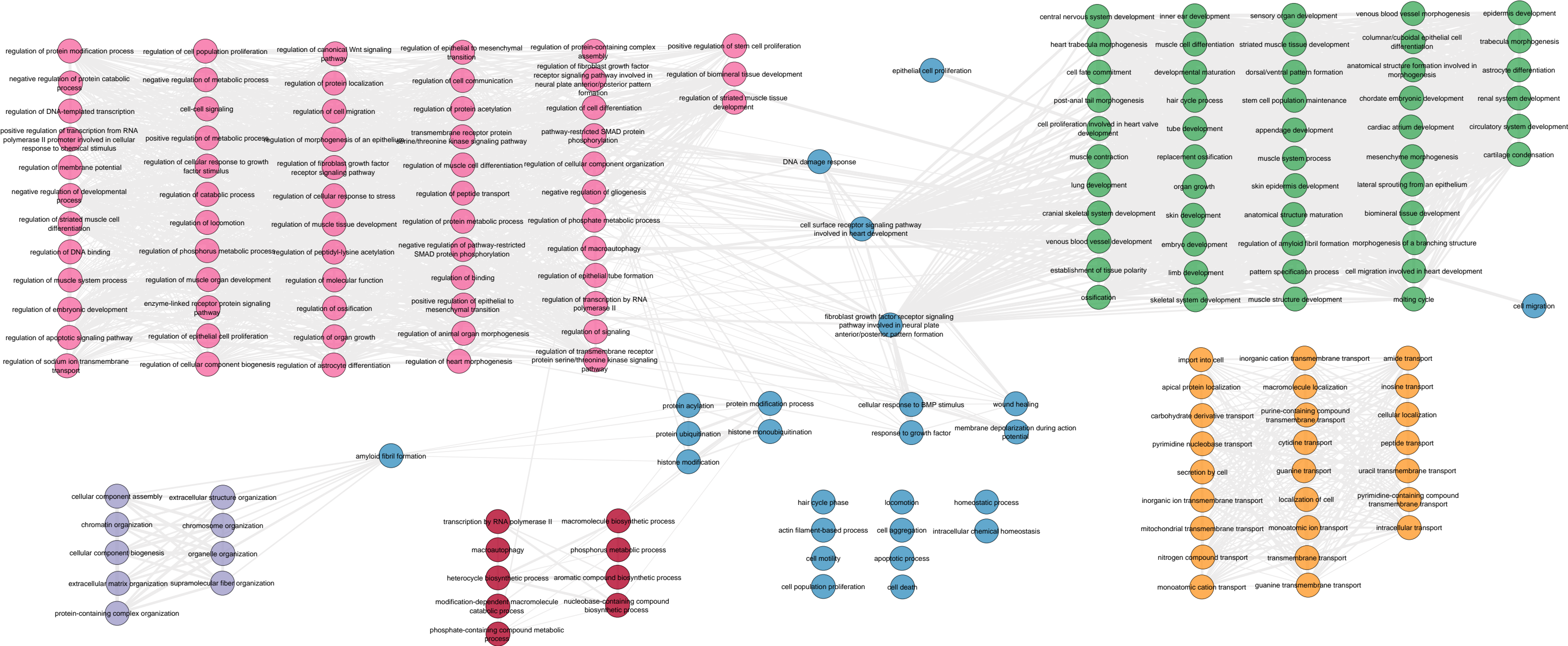

Supplement: Supplementary file 3 — Supplementary Material 3 [file 335_2025_10189_MOESM3_ESM.pdf]
